# Design and evaluation of a clinical competency committee

**DOI:** 10.1007/s40037-018-0490-1

**Published:** 2019-01-17

**Authors:** Marrigje E. Duitsman, Cornelia R. M. G. Fluit, Janiëlle A. E. M. van Alfen-van der Velden, Marieke de Visser, Marianne ten Kate-Booij, Diana H. J. M. Dolmans, Debbie A. D. C. Jaarsma, Jacqueline de Graaf

**Affiliations:** 1 Radboud University Medical Centre, Nijmegen, The Netherlands; 2 Erasmus Medical Centre, Rotterdam, The Netherlands; 3 Maastricht University, Maastricht, The Netherlands; 4 University Medical Centre Groningen, Groningen, The Netherlands

**Keywords:** Design-based research, Clinical competency committee, Assessment, Postgraduate medical education

## Abstract

**Introduction:**

In postgraduate medical education, group decision-making has emerged as an essential tool to evaluate the clinical progress of residents. Clinical competency committees (CCCs) have been set up to ensure informed decision-making and provide feedback regarding performance of residents. Despite this important task, it remains unclear how CCCs actually function in practice and how their performance should be evaluated.

**Methods:**

In the prototyping phase of a design-based approach, a CCC meeting was developed, using three theoretical design principles: (1) data from multiple assessment tools and multiple perspectives, (2) a shared mental model and (3) structured discussions. The meetings were held in a university children’s hospital and evaluated using observations, interviews with CCC members and an open-ended questionnaire among residents.

**Results:**

The structured discussions during the meetings provided a broad outline of resident performance, including identification of problematic and excellent residents. A shared mental model about the assessment criteria had developed over time. Residents were not always satisfied with the feedback they received after the meeting. Feedback that had been provided to a resident after the first CCC meeting was not addressed in the second meeting.

**Discussion:**

The principles that were used to design the CCC meeting were feasible in practice. Structured discussions, based on data from multiple assessment tools and multiple perspectives, provided a broad outline of resident performance. Residency programs that wish to implement CCCs can build on our design principles and adjust the prototype to their particular context. When running a CCC, it is important to consider feedback that has been provided to a resident after the previous meeting and to evaluate whether it has improved the resident’s performance.

**Electronic supplementary material:**

The online version of this article (10.1007/s40037-018-0490-1) contains supplementary material, which is available to authorized users.

**Table 1:** Minimal requirements of the Dutch Association of Paediatrics for the creation of a CCC

The program director must coordinate the formation and the evaluation process of a CCC
The CCC must meet at least twice a year
The judgment of the group members about the residents must be delivered on paper before the actual meeting to ensure its objectivity
The residents must provide relevant information about their progress before the meeting
It is optional for a program director to be present at the meeting. If they choose not to join the meeting, they must receive a written report of the meeting
It is optional to use input from people other than the members of the meeting
Residents’ progress of clinical competence must be judged using CanMEDs competencies, EPAs, exposure to clinical presentations and non-clinical duties

## What this paper adds

Clinical competency committees (CCCs) make informed decisions and provide feedback regarding resident performance. The process of creating and running a CCC should be considered carefully, since CCCs are advisory to the program directors as to whether residents are ready for unsupervised practice. It remains unclear how CCCs actually function in practice, and how their performance should be evaluated. Using a design-based research approach, this study evaluated theoretical design principles for implementing a CCC in a real-life setting. Residency programs can adapt these design principles to their particular context.

## Introduction

Group decision-making on the clinical progress of residents has emerged as an essential element in the evaluation process in postgraduate medical education. As of 2017, the Dutch Association of Paediatrics requires residency programs to develop clinical competency committees (CCCs) to assess the progress of residents [1]. A CCC consists of three or more members of the active teaching staff who make developmental recommendations to the program director and provide feedback to residents about their performance [2]. The process of creating and running a CCC must be considered carefully, since CCCs advise the program director as to whether residents are ready for unsupervised practice [2].

Groups can make better decisions than individuals by discussing existing data and sharing new, uniquely held information [3]. Group discussion can also increase detection of problematic performance in residents [4–7]. However, reality often falls short of these expectations [4, 8], which may jeopardize the validity of the judgment of resident performance.

The CCC literature [2, 4, 9–13] includes recommendations for starting a CCC [10, 12–14], reviews of group decision-making [4, 15, 16], and guidelines for creating and implementing a well-functioning CCC [2].

Given the importance of CCCs, it is essential to know how CCCs actually function in practice, and how their performance should be evaluated. Therefore, we conducted a design-based study, aiming [1] to develop and implement a prototype CCC meeting based on theoretical design principles and [2] to evaluate the prototype in a real-life setting.

## Methods

### Setting and participants

Our study was conducted at the Amalia Children’s Hospital in Nijmegen, the Netherlands. The Dutch Association of Paediatrics is the first Dutch accreditation agency to require their residency programs to develop CCCs. The Association set minimal requirements for designing a CCC (Tab. 1). Each training program is responsible for creating its own CCC, but many are unsure of how to best approach the process [9, 10, 12, 13].

The paediatrics training program comprises different subspecialties through which residents rotate. Each has a director responsible for resident training on the ward. Residents are expected to collect judgments using a prescribed minimum number of various assessment tools—e. g. mini clinical evaluation exercise (mini-CEXs), objective structured assessment of technical skills (OSATS)—in order to evidence their progress. The assessments have to be performed by supervisors (frontline assessors) at the workplace.

At the Amalia Children’s Hospital, 7 representatives of the subspecialties participated in the CCC, together with the program director and the vice-program director. All CCC members were clinicians who were also frontline workplace assessors. At the time of study, there were 24 residents training at this hospital: the CCC had to assess each of them at least twice a year.

We evaluated two CCC meetings that took place in October 2016 and February 2017. Both had the same members and chair. In the first, all 24 residents were discussed but, because of time pressure during the meeting, 12 were discussed in the second.

The CCC rated resident performance on CanMEDS competencies and entrustable professional activity (EPAs), and formulated feedback to help residents improve their performance. The CCC used previously gathered scores from multiple frontline workplace assessors and the judgments of individual CCC members of the overall performance of residents using a general assessment form. After the meetings, the resident’s daily supervisor delivered the CCC’s written feedback to the resident, supported by verbal feedback. All CCC members had been trained in providing feedback.

Prior to the first meeting, the program director discussed the purpose of the CCC, how to fill out the assessment forms, and how to interpret EPA levels and CanMEDS competencies with each member. She also discussed the purpose of the CCC with the residents. The program director had been trained in management and leadership skills but not specifically in managing group discussions.

Our institution waived ethical approval for this study, since ethical approval is not required for this type of research (implementation study and quality improvement of medical education). Our study was designed and performed in accordance with the Declaration of Helsinki; original data were treated confidentially and were only available to three researchers of the team (MD, MV, CF). All analyses were performed anonymously, participation was voluntary and written informed consent was obtained from all participants.

## Approach

We used a design-based research approach aimed at bridging gaps between theory and practice [17]. This approach focuses on the effect of an intervention in a real-life setting. Important principles of design-based research are that: the design of the intervention has to be based on theoretical principles; research is performed using design, evaluation and redesign cycles; evaluation of the intervention uses mixed methods; and designers and researchers with different expertise work together [18].

### Development of a prototype CCC meeting: three design principles

In the prototyping phase, the main researcher (MD) wrote a first draft of design principles for a CCC meeting based on theoretical perspectives on group assessment. After discussion with the research team, consensus was reached on three overarching principles: (1) data from multiple assessment tools and multiple perspectives, (2) a shared mental model and (3) structured discussion to reach consensus. Although the chosen design principles are not exhaustive, we aimed to include those of value to the CCC under study and limit the list of principles used.

Subsequently, MD, LF and JA developed a prototype CCC meeting. The prototype was designed to ensure consistent group decision-making in relation to resident performance, tailored to the demands of our specific context. The Table in the online Electronic Supplementary Material shows how the design principles were applied in the prototype. The three design principles are discussed in more detail below.

#### Data from multiple assessment tools and multiple perspectives

Group decisions on resident performance may be a valuable assessment method [19]. The group perspective is used to interpret various data to determine whether residents are ready for unsupervised practice. Critical for the quality of this review process is that each CCC member needs to provide a professional judgment on the resident’s performance [20] because, when multiple independent judgments are aggregated, value is created [21]. The value of group judgment increases with the number of independent judgments and different perspectives [21, 22]. Furthermore, sharing written information and assessment data before the meeting deepens the discussion [23].

Group decision-making is, therefore, ideally a combination of (1) the consideration of written information beforehand, i. e. multiple assessment by multiple frontline assessors, combined with written, independent judgments from CCC members of the overall performance of residents, and (2) discussing this information during the meeting and sharing and integrating new, uniquely held information.

#### Shared mental model

Effective group performance requires a common understanding of the task that has to be performed. This does not imply that members share the same opinion about issues discussed, but that group members hold common cognitive representations of task requirements and the teamwork that is involved [24]. Group members should have a common understanding of the purpose of the group meeting, how to interpret information, how judgmental decisions are made and what kind of teamwork and actions are needed to accomplish the task at hand [23, 24]. In groups without a shared mental model, optimal decision-making is impeded [4, 25].

#### Structured discussion

Good group decisions can be jeopardized by lack of discussion and by biases in information sharing [26]. An example is the potential for group members to discuss information already known to all group members rather than unshared information which is uniquely held by one person [27]. It is necessary to incorporate each member’s unshared information into the discussion to come to an optimal decision. [28–30]. Heterogeneous groups (composed of members with different characteristics) share more unshared information and different opinions than homogeneous groups [31]. Group leaders should structure the discussion in order to create optimal conditions for good group decision-making, facilitate the sharing of unshared information, avoid reaching agreement too soon and minimize social influence [24, 31, 32]. This can be done by summarizing the information to encourage more discussion or new points of view, letting the group members speak in a set order to ensure that all members have the opportunity to give their opinion and encouraging members to share divergent information [4, 33]. Group leaders should remain neutral during the CCC meeting, since their influence often dominates that of other members, which may hinder the best possible outcome [34]. Group size can also affect interaction between group members; a group size of 5–10 optimizes performance [35–37]. Finally, time pressure has to be avoided since it may minimize the time for discussion in order to come to an agreement faster [38].

### Evaluation of the CCC

To systematically study and structure the prototype CCC meeting, we evaluated the implementation in two cycles: the first cycle started in October 2016 and the second in February 2017. Evaluation of the first meeting resulted in some modifications of the prototype prior to the second meeting. These modifications are described in Tab. 2 and 3 and 4.

### Instruments

The following evaluation methods were used in each cycle:

- Observation of the meeting using a semi-structured observation guide (Appendix 1 of the online Electronic Supplementary Material);
- Individual, semi-structured interviews with all members of the committee (Appendix 2, online);
- Questionnaire with open-ended questions to be completed by all residents (Appendix 3, online).

The instruments were developed by MD, CF, JA and DJ. The questionnaire was administered to residents online.

### Data collection and analysis

In October 2016, the first CCC meeting was observed by two researchers (MD and CF), using the semi-structured observation guide. After the meeting, MD and CF discussed their findings, which were the same. Interviews were performed in the weeks following the meeting; questionnaires were sent 4 weeks after the meeting to ensure that CCC members had enough time to deliver feedback to residents. In February 2017, one researcher (MV) observed the second CCC meeting. Since the two observers of the first CCC meeting found the same results, we decided to use one observer for the second meeting. She conducted the interviews in the week following the meeting and the questionnaires were sent 4 weeks after the meeting. We analyzed the field notes from the observations, the interviews and the questionnaires by conducting a content analysis, keeping the design principles in mind [39, 40]. Two researchers (MD and CF) individually analyzed the data and discussed their findings until consensus was reached. The resulting codes and themes were, if possible, organized on the basis of the design principles and remaining themes categorized in an additional category. The themes were discussed by the entire research team and the results were translated into modifications to the design of the CCC meeting that were to be implemented in the next meeting. Although our sample size of CCC members and residents was relatively small, we reached thematic saturation since the final interviews and questionnaires did not provide any additional information.

### Research team

Our interdisciplinary research team added to the rigour and quality of the research. The observers, interviewers and researchers who analyzed the data were not members of the CCC. JA was the program director and chaired the CCC meetings. Consequently, she had inside information about all CCC members and residents. This underlined the need for continuous team reflexivity. JG and MKB, who were program directors of another medical specialty, reflected on the findings based on their experience in judging resident performance. MD, MV, CF, DD and DJ looked at the results from both an educationalist’s and a researcher’s perspective.

## Results

The main results of the evaluation of the two cycles will be presented together, because we consider this research to be an overarching cyclic process of design (Tab. 2, 3 and 4).

### Observation of the meetings

In the first meeting, the CCC discussed 24 residents in 150 min; however, its members felt time pressure towards the end of the meeting. Therefore, in the second meeting, 12 residents were discussed in 120 min ensuring adequate time for discussion of each resident. The group leader encouraged CCC members to speak and actively asked for additional information, which in turn enhanced constructive discussion. During the first meeting, CCC members had different mindsets about EPA levels and CanMEDS competencies. In the second meeting there were still discussions about how to assess the performance of residents, however, less than in the first meeting, showing that the CCC started to acquire a shared understanding. The group leader asked the CCC members to speak in a set order to ensure that they all shared their opinions. All discussions ended by formulating feedback to residents, comprising both positive feedback and three points for improvement. Finally, in the second meeting, the CCC did not evaluate whether the resident had addressed the previously identified developmental needs.

### Interviews with CCC members

All CCC members perceived that they had gained a richer picture of the residents by discussing their performance. They had also identified problematic and excellent performance that they would not have recognized individually. Two members were hesitant to provide negative judgments on residents in the first meeting because they were afraid to harm the resident’s career. All CCC members reported that they had learned different approaches to assessing and supervising residents from their colleagues. Some CCC members had not discussed resident performance with their colleagues prior to the meeting, whereas others had, including nurses and paramedical staff. The latter felt that they had gained a more grounded understanding of resident performance.

### Resident questionnaires

After the first CCC meeting, the response rate for the questionnaire among residents was 75% (18 out of 24) and after the second 50% (6 out of 12). In general, residents felt that their performance had been carefully discussed and carefully judged. They appreciated the group judgments, although some residents were disappointed about the feedback. They felt that the supervisor who provided the feedback mitigated the message by stating that not all members had agreed. Therefore, they did not always take the feedback seriously. Furthermore, residents considered the time span of 2 months between receiving the feedback after a CCC meeting and the moment they had to hand in information for the next meeting too short to collect evidence for performance improvement. Tab. 4 shows the main results of the questionnaires and modifications to the prototype CCC meeting that had been made accordingly.

## Discussion

These CCC meetings facilitated structured discussions about resident performance that were informed by multiple assessment data and multiple opinions, resulting in well-informed judgments. The group discussions led to identification of problematic and excellent performance that would not have been recognized otherwise. The discussion time was the same for all residents and each discussion resulted in a plan of action containing positive feedback and three points for improvement. A notable finding was that the CCC did not reflect on feedback provided to residents after the previous meeting. There was uncertainty among CCC members about how to interpret EPA levels and CanMEDS competency levels. Residents were not always satisfied with their feedback.

Our finding that some CCC members realized that they had not always been able to identify excellent or problematic performance on their own supports the use of group decision-making and that group assessment reduces the halo effect compared with individual judgment [6, 7]. An important part of group decision-making is to share uniquely held information that is unknown to others [28, 41], which was facilitated by our structured group discussions. It also resulted in an action plan for each resident suggesting adoption of a developmental approach. Hauer and colleagues [9] found that CCCs focused on identifying problematic or struggling residents rather than using a developmental approach. In our opinion, allotting a fixed amount of discussion time for each resident, formulating a concise summary of the feedback and an action plan with improvement points for each resident builds on a developmental approach. Nevertheless, the CCC did not reflect on feedback provided after the previous CCC meeting which we consider hampered the developmental approach. It can only be confirmed that residents learn from feedback when they act on it, which completes the feedback loop [42, 43]. Therefore, residents should provide evidence that the previously identified developmental needs have been addressed and CCCs should evaluate whether their performance has been improved.

Feedback that enhances performance is feedback that is valued [43–45]: not all residents took the feedback seriously, which jeopardizes its learning potential. Residents felt that feedback providers had mitigated the message (e. g. ‘The other CCC members said …’). Therefore, they wondered how seriously to take the feedback. While such mitigation can help secure harmonious relationships, it can also create confusion or misunderstanding [44, 46].

The development of a shared mental model was an underlying principle. CCC members used different standards to judge the performances of residents and had different notions about it. Observation of the discussions during the meetings indicates that the CCC members learned from each other. The interview results supported this view. After the first meeting, the group leader explained the assessment criteria once more to the team members. During the second meeting, we noticed more common understanding of the judgment criteria, supported by the training given and through the CCC’s team interaction [47, 48].

A limitation of our study may be that the observations during and interviews after the first meeting were performed by different researchers than those of the second meeting. This could have led to slightly different observations and different interviewing styles between researchers. However, we used semi-structured observation and interview guides and rigorously analyzed the data, aiming to minimize possible differences.

Future research should focus on CCCs in different contexts, on optimizing feedback to residents, and on closing the feedback loop.

Our design principles, namely (1) data from multiple assessment tools and multiple perspectives, (2) a shared mental model and (3) structured discussion to reach consensus, can be used to implement a CCC that employs a developmental approach to assessment. It is important to evaluate CCC performance. A promising way of doing this is implementing a cyclic process of evaluation. However, this kind of evaluation is time-consuming and may not be feasible in every setting.

CCCs should complete the feedback loop by reflecting on feedback provided to residents after the previous meeting and reviewing whether residents have acted on it to improve their performance.

**Table 2:** Main observations

Design principles	Cycle #1	Modification	Cycle #2
1. Multiple assessment data and multiple perspectives	Residents delivered multiple assessment data	No modification needed	Residents delivered multiple assessment data
2. Shared mental model	Discussions about the way of assessing residents	GL gave the CCC members an additional explanation of the EPA levels and CANMED levels	Still some discussions, but less than in first meeting
3. Interaction during the meeting	Time pressure	Instead of twice a year 24 residents, the frequency changed to 4 times a year 12 residents	No time pressure
	Group leader (GL) structured the meeting	No modification needed	GL structured the meeting
	No equal participation of CCC members	We advised the GL to actively invite silent members to speak	GL actively invited silent members to speak up; more equal participation of the members
Extra	–	–	No feedback loop

**Table 3:** Main results from the interviews

Design principles	Cycle #1	Modification	Cycle #2	Quotes
1. Multiple assessment data and multiple perspectives	Not all members discussed resident performance with their colleagues before the meeting	The group leader asked the CCC members to consult colleagues before the meeting	More members consulted their colleagues and said they formed a broader picture about resident performance by doing so	M6: ‘I did not consult my colleagues before the meeting, but I am going to schedule time before the next meeting to do so, because I think it is valuable’
2. Shared mental model	Members learned from each other about different approaches of assessing from colleagues	No modification needed	Members learned from each other about different approaches of assessing from colleagues	M6: ‘All supervision levels must be the same at every ward. So that we assess residents in the same way’
3. Interaction during the meeting	Safe atmosphere	No modification needed	Safe atmosphere	M3: ‘There was a really good atmosphere, I had the feeling I could say everything’
	Some members were hesitant to give a negative opinion	The CCC discussed this hesitation and discussed the need to give negative opinions when necessary	The members were more comfortable in giving a negative opinion when necessary	M2: ‘I cannot do that, can I? To somebody who is such a nice person, to give, to (…) I have the feeling I cannot do that!’ [give a negative opinion]
Extra	Broad and rich picture about the performance of residents	No modification needed	Broad and rich picture about the performance of residents	M1: ‘I have a broader image of the resident, especially because I now know his extracurricular activities. We did not know that before the introduction of the CCC’
	They were concerned about the extent to which private matters of the resident should be discussed	It was decided to ask the permission of residents before the meeting to discuss private matters	Private matters were only discussed when residents gave their permission	M4: ‘This level of feedback and assessment cannot be reached with feedback on paper. Now we were able to ask questions about somebody’s opinion and discuss this*’*

**Table 4:** Main results from the questionnaires

Design principles	Cycle #1	Modification	Cycle #2	Quotes
1. Multiple assessment data and multiple perspectives	All residents were able to deliver multiple assessment data prior to the CCC	No modification needed	All residents were able to deliver multiple assessment data prior to the CCC	Q14: ‘Collecting multiple data points and reviewing those again is a good thing. It made me realise again what my points for improvement are’
Extra	Residents felt that their performance was seriously discussed in the CCC	None	Residents felt that their performance was seriously discussed in the CCC	Q8: ‘It creates a broad picture of you as a person, as a doctor, about your strengths and weaknesses and not just a picture from one rotation or from one supervisor’
	All residents thought that there was too little time between the meeting, the feedback they got back and preparation for the next meeting	Feedback was delivered as soon as possible after the second meeting	They were satisfied with the early delivery of feedback and felt they had more time to work on the feedback before the next meeting	Q6: ‘The second CCC was too soon after the feedback from the first CCC. Therefore, the feedback from the second meeting was still the same’
	Some residents were not satisfied with the content of the feedback they received after the meeting	None (because this was outside the scope of our study)	Some residents were not satisfied with the content of the feedback they received after the meeting	Q11: ‘Feedback should be founded on concrete examples of behaviour, not on vague remarks like: ‘I had the feeling that […]’ Then it is just one person’s opinion
				Q3: ‘The feedback was exactly the same as during the latest rotation. I did not feel like the group added something to the opinion of my most recent supervisor’

## Caption Electronic Supplementary Material

Table 1. Prototype CCC

Appendix 1. Semi-structured observation guide

Appendix 2. Semi-structured interview guide CCC members

Appendix 3. Questionnaire residents
