# Supplementary material for: Design and evaluation of a clinical competency committee
Source: Perspect Med Educ. 2019 Jan 17;8(1):1–8. doi: 10.1007/s40037-018-0490-1 (PMC6382624; doi:10.1007/s40037-018-0490-1)
Supplement: Supplementary file 1 — Table 1. Prototype CCC [file 40037_2018_490_MOESM1_ESM.docx]

**Table 1. Prototype CCC**

| **Design principles** | **Prototype (specific design for our context)** |
| --- | --- |
| 1. Multiple assessment data and perspectives | **Residents must deliver multiple assessment data** and other relevant information about their progress. Furthermore, they have the option to ask a specific question to the CCC.  Ideally, CCCs consist of clinicians as well as nurses and paramedics to ensure the heterogeneousness of the group to assemble divergent opinions. In our specific situation this was not feasible in practice. As a solution, all **CCC members should consult with their colleagues before the meeting (colleague clinicians, nurses or paramedics)** to ensure that divergent experiences with the resident are taken into account.  **Members must fill out the assessment forms beforehand** and discuss these during the meeting. |
| 2. Shared mental model | Before the first meeting **the program director should explain the purpose of the meeting** to the CCC members and explain to the members how to fill out the assessment forms. The PD also explains the purpose and process of the CCC to the residents.  **Information that the residents delivered is sent 4 weeks prior to the meeting to all the members and the written assessment forms are filled in by the CCC members were sent two weeks prior to the meeting**. |
| 3. Interaction during the meeting | **CCC consists of 9 members**  The CCC consists of the program director, the assistant program director and 7 directors of different subspecialties  **The program director is the group leader (GL)**  The group leader should not bring in information about the resident to avoid domination of opinion, and also to be able to focus on managing the discussion. In our context, the vice-program director is also part of the CCC and was therefore able to bring in uniquely held information by the PD.  **Members should share their personal experiences with the group during the meeting**  **GL must structure the meeting**  The group leader must be aware to let members speak in a set order; to give every member the opportunity to speak; to encourage members to speak, give examples and share (different) opinions; to summarize the information to elicit more discussion and new points of view; and to actively ask for more information  **The group leader should carefully guard time** to avoid time pressure. |
