# Supplementary material for: Design and evaluation of a clinical competency committee
Source: Perspect Med Educ. 2019 Jan 17;8(1):1–8. doi: 10.1007/s40037-018-0490-1 (PMC6382624; doi:10.1007/s40037-018-0490-1)
Supplement: Supplementary file 2 — Appendix 1. Semi-structured observation guide [file 40037_2018_490_MOESM2_ESM.docx]

**Appendix 1. Semi-structured observation guide**

| **Characteristics of CCC** |  |
| --- | --- |
| - How many members? |  |
| - Who are the members? (function) |  |
| - Do all the members participate? |  |
| **Information sharing** |  |
| - What information do they share? |  |
| - Do the members listen to each other? |  |
| - Extra |  |
| **Group leader** |  |
| **Structuring the meeting** |  |
| - In what order do members speak? - Does the group leader give every member have the opportunity to speak? |  |
| - In what order are the residents presented? |  |
| - Do the members utter divergent opinions about the residents? - Does the group leader elicit divergent opinions? |  |
| - Does the group leader encourage members to give examples, opinions? |  |
| - Does the group leader encourage discussion? |  |
| - Does the group leader summarize the information? |  |
| - Does the group leader ask for more information in an active way? |  |
| **Time** |  |
| Does every resident got the same amount of discussion time? |  |
| Is there time pressure? |  |
| **Extra** |  |
| Room for other striking features |  |
