# Supplementary material for: Design and evaluation of a clinical competency committee
Source: Perspect Med Educ. 2019 Jan 17;8(1):1–8. doi: 10.1007/s40037-018-0490-1 (PMC6382624; doi:10.1007/s40037-018-0490-1)
Supplement: Supplementary file 3 — Appendix 2. Semi-structured interview guide CCC members [file 40037_2018_490_MOESM3_ESM.docx]

**Appendix 2. Semi-structured interview guide CCC members**

**Preparation**

- Can you tell something about how you prepared for the meeting?
- How did you form a judgment about the performance of the residents?
  - Did you consult or discuss this with colleagues?
- What do you think about the preparation for the meeting?

**The meeting**

- Were you able to say what you wanted to say about the residents?
  - If yes, what made this possible?
  - If no, what were the reasons?
- Did you hear new / surprising information about residents?
- Did you agree with the final judgment and feedback to the resident? If not, why? Did you express this disagreement?
- What did you think about the performance of the group leader?
- What do you think about the quality of the meeting?

**Extra**

- What are the benefits of a CCC meeting?
- What are the disadvantages of a CCC meeting?
- What would you like to see different next meeting?
