# Supplementary material for: Design and evaluation of a clinical competency committee
Source: Perspect Med Educ. 2019 Jan 17;8(1):1–8. doi: 10.1007/s40037-018-0490-1 (PMC6382624; doi:10.1007/s40037-018-0490-1)
Supplement: Supplementary file 4 — Appendix 3. Questionnaire residents [file 40037_2018_490_MOESM4_ESM.docx]

**Appendix 3. Questionnaire residents**

1. What did you think about your preparation prior to the CCC meeting?

Did you miss something in the preparation? (e.g. information about yourself you wanted to add?)

Did you find some things unnecessary in the preparation?

2. You were asked to formulate a specific question to the CCC; did you find this valuable? Can you explain your answer?

3. Do you think the question to the CCC must become obligatory? Can you explain your answer?

4. What are the benefits of the CCC meeting?

5. What are the disadvantages of the CCC meeting?

6. What do you think about the feedback you received after the CCC meeting?

Did you receive new/surprising feedback?

Did you receive valuable feedback?

Did you recognize yourself in the feedback you received?

Did you feel that the feedback was carried by the whole CCC? Can you explain your answer?

7. Does the CCC meeting add something to your training? If so, can you explain in what way? If no, can you explain why?

8. How do you think that CCC members must handle private information they have about your personal situation?

9. Do you have suggestions for the next meeting?
